# Social Factors, Age, and Health at Time of Dementia Diagnosis

**DOI:** 10.1001/jamanetworkopen.2024.61117

**Published:** 2025-02-21

**Authors:** Mozhu Ding, Katharina Schmidt-Mende, Karin Modig

**Affiliations:** 1 Unit of Epidemiology, Institute of Environmental Medicine, Karolinska Institutet, Stockholm, Sweden; 2 Academic Primary Health Care Centre, Stockholm Region, Stockholm, Sweden; 3 Division of Family Medicine and Primary Care, Department of Neurobiology, Care Sciences and Society, Karolinska Institutet, Huddinge, Sweden

## Abstract

**Importance:**

A timely diagnosis is crucial in ensuring high-quality care for individuals with dementia. Comparing age and health status at the time of dementia diagnosis across population subgroups can provide insights into vulnerable groups that may experience delayed diagnosis and/or a poorer prognosis.

**Objective:**

To examine how age and health at the time of dementia diagnosis vary across sociodemographic subgroups.

**Design, Setting, and Participants:**

A nationwide cross-sectional study of individuals with an incident dementia diagnosis from either specialist care or who were dispensed antidementia drugs, identified from the Swedish National Patient Register and the Prescribed Drug Register between January 2018 and December 2022.

**Exposures:**

Sociodemographic status at the time of dementia diagnosis was assessed through linkage with national registers and included education, place of birth, living arrangement, family status, and family disposable income.

**Main outcomes and measures:**

Health status was assessed by calculating the number of dispensed medications during the year preceding dementia diagnosis, as well as the Charlson Comorbidity Index (CCI) and Hospital Frailty Risk Score (HFRS) using specialist diagnoses during the preceding 5 years.

**Results:**

Of the 107 707 individuals with a dementia diagnosis, 61 127 (56.8%) were women and the mean (SD) age at diagnosis was 82.0 (7.7) years. Individuals with lower education, living alone or in a care home, or without a close relative were a mean (SD) of 3.1 (7.3) years older when diagnosed with dementia compared with their counterparts. At the time of diagnosis, individuals who had lower than high school education had higher CCI (odds ratio [OR], 1.11; 95% CI, 1.07-1.15) and more medications (OR, 1.38; 95% CI, 1.33-1.44) compared with those with university education. Age and health status at dementia diagnosis did not differ significantly by place of birth or income.

**Conclusions and relevance:**

This nationwide register-based study indicated that clinical diagnosis of dementia may be significantly delayed among individuals with low education, those living alone or in a care home, or those without a close relative. These groups also had poorer health at the time of dementia diagnosis, which may impact their prognosis negatively. Future efforts should focus on improving health services to better address the diagnostic and care needs of these vulnerable subgroups.

## Key Points

**Question:**

How do age and health status at the time of dementia diagnosis differ across sociodemographic subgroups in Sweden?

**Findings:**

In this nationwide cross-sectional study of 107 707 individuals with a first-ever dementia diagnosis, people with lower education, living alone, or without a close relative were a mean of 3.1 years older and with poorer health status than their counterparts at the time of their dementia diagnosis.

**Meaning:**

These findings suggest clinical diagnosis of dementia may be significantly delayed among sociodemographically disadvantaged subgroups.

## Teaser

This cross-sectional study evaluates how age and health at the time of dementia diagnosis vary across sociodemographic subgroups in Sweden.

## Introduction

Receiving a dementia diagnosis can be devastating for both patients and their families, and a timely diagnosis is crucial for ensuring high-quality care^1,2^ and interventions that can help maintain quality of life.^3,4^ Missed or delayed diagnoses can exacerbate disability, reduce intervention effectiveness, and increase caregiver burden and health care costs.^5,6^

Strikingly, approximately 50% of older adults with dementia remain undiagnosed or receive a late diagnosis.^7,8^ Because current evidence does not support routine dementia screening, diagnostic assessment of dementia is mostly initiated in response to the patient’s complaints or concerns raised by relatives or caregivers.^9^ Thus, the timing of a dementia diagnosis is influenced by both the underlying risk of dementia and care-seeking behaviors. While underdiagnosis of dementia in certain subgroups has been previously reported,^8,10^ the relationship between sociodemographic factors and the timing of a dementia diagnosis is less studied. While factors such as low education and social isolation are linked to a higher risk and earlier onset of dementia,^11,12^ low education, living alone, or low income are associated with a reduced likelihood of receiving a formal dementia diagnosis, which may result in diagnostic delays.^13,14,15,16^

Current evidence on delays in dementia diagnosis primarily stems from local screening cohorts, often limited by small sample sizes and selected populations.^17,18,19,20^ Although large-scale administrative data are increasingly used to study health outcomes of dementia, there are no nationwide studies in Sweden, and few globally,^21^ that investigate how age and health differ at the time of diagnosis across population subgroups. Health status of individuals with dementia, such as frailty index, has been suggested as an indicator of dementia severity in administrative data,^22,23,24^ and higher comorbidity burden has been associated with faster dementia progression and worse survival.^25^ By comparing age and health status at the time of dementia diagnosis across sociodemographic subgroups, we can gain insights into the relative timing of dementia diagnosis and identify subgroups that may experience greater diagnostic delays and risk of a poorer prognosis. Swedish administrative health registers offer a unique opportunity to describe this for the total population. The registers can identify all diagnoses made in specialist care and all dispensed drugs. The national guidelines for dementia treatment recommend prescription of antidementia drugs following a diagnosis of Alzheimer disease (AD) or mixed dementia,^26^ and uptake have been reported to be 85% among individuals with an AD diagnosis.^27^ Therefore, a combination of specialist care and antidementia drugs provide good coverage of individuals with a dementia diagnosis, although for subtypes other than AD and mixed dementia there might still be some underestimation.

This study aimed to describe age and health status at the time of dementia diagnosis across sociodemographic subgroups. We hypothesized that subgroups that were disadvantaged in sociodemographic status (ie, education, living arrangement, family status, place of birth [Sweden or elsewhere], or income) would be older and in worse health when diagnosed with dementia, suggesting a greater diagnostic delay in these groups.

## Methods

### Study Population

Data were derived through multiple Swedish national registers covering all individuals in Sweden born before 1960 and followed up until end of 2022. Information was linked together through a unique personal identification number assigned to each individual residing in Sweden. Informed consent is not required for register-based research in Sweden. This study was approved by the Regional Ethics Committee in Stockholm, follows the Strengthening the Reporting of Observational Studies in Epidemiology (STROBE) reporting guideline for cross-sectional studies, and was performed in accordance with the 1964 Declaration of Helsinki and its later amendments.

We identified 107 707 incident dementia diagnoses between January 1, 2018, and December 31, 2022. Diagnosis date was defined as the date of first specialist care diagnosis in the National Patient Register (NPR) or dispensed antidementia medication in the Prescribed Drug Register (PDR), whichever came first. The NPR contains inpatient care with nationwide coverage since 1987 and specialist outpatient care since 2001; dates and discharge diagnoses of each visit are coded according to the *International Statistical Classification of Diseases and Related Health Problems, Tenth Revision (ICD-10)*. We used *ICD-10* codes F00, F01, F02, F03, F05.1, and G30 to identify dementia diagnosis. The PDR contains nationwide information on all dispensed medications since 2005, coded using the Anatomical Therapeutic Chemical (ATC) system. ATC code N06D was used to identify antidementia drugs. Antidementia drugs could serve as a proxy of dementia cases diagnosed in primary care only, which we did not have access to. It was shown that 95% of dementia diagnoses in Stockholm could be captured by specialist care diagnosis or antidementia drug use.^8^

### Demographic and Sociodemographic Variables

Data on date of birth, country of birth, sex, highest attained education, and family disposable income were retrieved from the Longitudinal Integrated Database for Health Insurance and Labour Market Studies register. Education was categorized according to years of formal schooling into lower than high school (≤9 years), high school (10-12 years), and university (≥13 years). Family disposable income in the year preceding dementia diagnosis was categorized into tertiles based on the age group-specific tertile. Information on living arrangements in the year preceding dementia diagnosis was retrieved from the Dwelling Register, which provides data on cohabitation status, as well as the Swedish Social Service Register, which provides data on care home services for older adults. Individuals were categorized as living alone at home, living with someone at home, and living in a care home. Data on family status were derived from the Multigeneration Register. Individuals cohabiting or having adult children were classified as having a close relative; individuals living alone or in a care home without adult children were classified as not having a close relative.

### Health Status at the Time of Dementia Diagnosis

Health status was measured through Charlson Comorbidity Index (CCI), Hospital Frailty Risk Score (HFRS), and number of prescribed medications. The calculation of CCI and HFRS was based on diagnoses identified in the NPR in the 5 years preceding dementia diagnosis. CCI was calculated following the methods developed by Ludvigsson et al,^28^ an adapted version of the CCI to be used in Swedish registers. The corresponding *ICD-10* codes used to identify diseases in the CCI are presented in eTable 1 in Supplement 1. The HFRS was calculated following the methods developed by Gilbert et al^29^ which used 109 *ICD-10* codes weighted and summed to create a frailty score (eTable 2 in Supplement 1). Because CCI and HFRS were based on diagnoses before dementia diagnosis, dementia did not contribute to the calculation of these scores. From the PDR, the number of dispensed medications was calculated by counting the number of drug classes at the third ATC level (eg, C07A) prescribed during the 1-year period before the dementia diagnosis.

### Statistical Analysis

Mean and SD of age, CCI, HFRS, and number of medications at the time of dementia diagnoses were compared across sociodemographic subgroups. Logistic regression models were used to estimate the odds ratios (OR) and 95% CI of HFRS greater than 0, CCI of 1 or more, and 5 or more medications at the time of dementia diagnosis by sociodemographic factors, adjusting for age at diagnosis and sex. Sociodemographic factors were not adjusted for each other at the same time due to risk of collinearity. Moreover, CCI, HFRS, and number of medications were treated as categorical variables and compared across sociodemographic subgroups using multinomial logistic regression models. CCI was categorized into 0, 1 to 2, 3 to 4, and 5 or more; HFRS into 0, 1 to 4, 5 to 14, and 15 or more; and number of medications into 0, 1 to 4, 5 to 9, and 10 or more. Several sensitivity analyses were performed: (1) we excluded individuals identified by drug use alone to account for a potentially earlier diagnosis than the date of prescription; (2) we restricted the dementia identification period to January 1, 2018, to December 31, 2019, to reduce potential influence of the COVID-19 pandemic on clinical practice; and (3) we stratified the analyses by urban and rural area to account for regional variations in care use.

## Results

Of the 107 707 individuals with an incident dementia diagnosis during 2018 to 2022, 61 127 (56.8%) were women and the mean (SD) age at diagnosis was 82.0 (7.7) years. A total of 28 906 individuals (26.8%) at dementia diagnosis had lower than high school education, 39 582 (36.8%) were living alone at home, 57 515 (53.4%) were without a close relative, and 13 950 (13%) were born outside of Sweden. Women at dementia diagnosis were more likely than men to be living alone at home (27 673 [45.3%] vs 11 909 [25.6%]), have a close relative (35 001 [57.3%] vs 14 726 [31.6%]), and have a family income in the lower tertile (25 700 [42.0%] vs 10 791 [23.2%]) (Table 1).

**Table 1.** Characteristics of Individuals at the Time of Dementia Diagnosis, 2018 to 2022

Characteristic	Individuals, No. (%)		
	Total (N = 107 707)	Men (n = 46 580)	Women (n = 61 127)
Age, mean (SD)	82.0 (7.7)	81.0 (7.5)	83.0 (7.7)
Education^a^			
Lower than high school	28 906 (26.8)	11 994 (25.8)	16 912 (27.7)
High school	31 500 (29.3)	13 374 (28.7)	18 126 (29.7)
University or above	17 743 (16.5)	7694 (16.5)	10 049 (16.4)
Living arrangement			
With someone at home	49 594 (46.1)	27 941 (60.0)	21 653 (35.4)
Alone at home	39 582 (36.8)	11 909 (25.6)	27 673 (45.3)
In a care home	18 531 (17.2)	6730 (14.5)	11 801 (19.3)
Family status			
With a close relative	49 727 (46.2)	14 726 (31.6)	35 001 (57.3)
Without a close relative	57 515 (53.4)	31 607 (67.9)	25 908 (42.4)
Place of birth			
Sweden	93 757 (87.0)	40 943 (87.9)	52 814 (86.4)
Outside of Sweden	13 950 (13.0)	5637 (12.1)	8313 (13.6)
Family disposable income			
Higher tertile	35 282 (32.8)	19 362 (41.6)	15 920 (26.0)
Middle tertile	35 934 (33.4)	16 427 (35.3)	19 507 (31.9)
Lower tertile	36 491 (33.9)	10 791 (23.2)	25 700 (42.0)
Frailty score, mean (SD)	4.8 (5.5)	5.2 (5.7)	4.6 (5.2)
Charlson Comorbidity index, mean (SD)	1.2 (1.7)	1.5 (1.8)	1.0 (1.5)
No. of prescribed medications, mean (SD)	7.5 (4.2)	7.4 (4.1)	7.6 (4.2)

^a^ Education level was missing for 29 558 individuals (27.4%).

Table 2 shows age at dementia diagnosis by sociodemographic factors. Among all individuals with dementia diagnoses, those with education lower than high school were a mean (SD) of 3.1 (7.3) years older when receiving a dementia diagnosis compared with those with university education (mean [SD] age 82.7 [7.3] vs 79.6 [7.4]). Similarly, individuals who were living alone and individuals without a close relative were 3.0 and 3.6 years older than their counterparts when diagnosed with dementia. However, age at dementia diagnosis did not differ substantially by place of birth (mean [SD] age 82.3 [7.6] years for those born in Sweden vs 81.1 [7.8] years for those born elsewhere) or by family income.

**Table 2.** Age and Health Status at the Time of Dementia Diagnosis Stratified by Sociodemographic Factors

Sociodemographic factor	Age at diagnosis, mean (SD)	HFRS			CCI			Prescribed medications		
		Mean (SD)	HFRS>0, No. (%)	OR (95% CI) of HFRS>0^a^	Mean (SD)	CCI≥1, No. (%)	OR (95% CI) of CCI≥1^a^	Mean (SD)	Medications ≥5, No. (%)	OR (95% CI) of medications ≥5^a^
Education level										
University	79.6 (7.4)	4.5 (5.1)	13 524 (76.1)	1 [Reference]	1.0 (1.5)	7839 (44.2)	1 [Reference]	6.6 (4.1)	11 768 (66.3)	1 [Reference]
High school	80.1 (7.5)	4.4 (5.2)	23 201 (73.7)	0.87 (0.84-0.91)^b^	1.1 (1.6)	14 469 (46.0)	1.07 (1.03-1.11)^b^	7.0 (4.1)	22 321 (70.9)	1.22 (1.17-1.27)^b^
Lower than high school	82.7 (7.3)	4.4 (5.2)	21 000 (72.7)	0.82 (0.78-0.86)^b^	1.1 (1.6)	13 876 (48.0)	1.11 (1.07-1.15)^b^	7.4 (4.1)	21 773 (75.3)	1.38 (1.33-1.44)^b^
Living arrangement										
With someone at home	79.9 (7.1)	4.3 (5.1)	36 680 (74.0)	1 [Reference]	1.2 (1.7)	24 297 (49.0)	1 [Reference]	7.0 (4.1)	35 047 (70.7)	1 [Reference]
Alone at home	82.9 (7.5)	4.7 (5.3)	29 702 (75.0)	1.14 (1.12-1.15)^b^	1.2 (1.6)	19 153 (48.3)	1.07 (1.06-1.08)^b^	7.1 (4.1)	28 585 (72.2)	0.99 (0.96-1.01)
In a care home	86.6 (7.3)	6.6 (6.4)	15 332 (82.8)	1.56 (1.54-1.59)^b^	1.4 (1.8)	10 524 (56.8)	1.20 (1.19-1.22)^b^	9.5 (3.9)	16 790 (90.6)	3.20 (3.03-3.38)^b^
Family status										
With a close relative	80.5 (7.2)	4.6 (5.3)	43 045 (74.8)	1 [Reference]	1.3 (1.7)	28 751 (50.0)	1 [Reference]	7.2 (4.2)	41 686 (72.5)	1 [Reference]
Without a close relative	84.1 (7.7)	5.1 (5.6)	38 402 (77.3)	1.17 (1.15-1.18)^b^	1.2 (1.7)	25 079 (50.4)	1.05 (1.04-1.06)^b^	7.8 (4.2)	38 507 (77.4)	1.14 (1.11-1.17)^b^
Place of birth										
Sweden	82.3 (7.6)	4.8 (5.4)	71 100 (75.8)	1 [Reference]	1.3 (1.8)	47 088 (50.3)	1 [Reference]	7.5 (4.1)	69 948 (74.6)	1 [Reference]
Outside of Sweden	81.1 (7.8)	4.9 (5.6)	10 614 (76.0)	1.03 (0.99-1.07)	1.2 (1.7)	6888 (49.4)	1.03 (1.02-1.04)^b^	7.7 (4.4)	10 474 (75.1)	1.09 (1.05-1.14)^b^
Family disposable income										
Higher tertile	81.8 (7.7)	4.9 (5.4)	27 207 (77.1)	1 [Reference]	1.3 (1.7)	17 820 (50.5)	1 [Reference]	7.2 (4.1)	25 523 (72.3)	1 [Reference]
Middle tertile	82.2 (7.5)	4.8 (5.5)	27 260 (75.9)	0.94 (0.91-0.97)^b^	1.3 (1.7)	18 288 (50.9)	1.06 (1.03-1.09)^b^	7.5 (4.2)	27 077 (75.4)	1.23 (1.19-1.28)^b^
Lower tertile	82.4 (7.8)	4.8 (5.5)	27 247 (74.7)	0.91 (0.88-0.94)^b^	1.2 (1.7)	17 866 (49.0)	1.05 (1.03-1.10)^b^	7.7 (4.3)	27 822 (76.2)	1.16 (1.12-1.20)^b^

Abbreviations: CCI, Charlson Comorbidity Index; HFRS, Hospital Frailty Risk Score; OR, odds ratio.

^a^ ORs are adjusted for age at diagnosis and sex.

^b^ *P* < .001.

Table 2 and the Figure show the health status at dementia diagnosis by sociodemographic factors. At the time of dementia diagnosis, people with high school or lower than high school education were more likely to have a CCI of 1 or higher (OR, 1.07; 95% CI, 1.03-1.11; OR, 1.11 and 95% CI, 1.07-1.15, respectively) and 5 or more prescribed medications (OR, 1.22; 95% CI, 1.17-1.27 and OR, 1.38, 95% CI, 1.33-1.44, respectively) compared with those with university education (Table 2). They were also more likely to have a higher proportion of CCI categories 1 to 2, 3 to 4, and 5 or more, as well as 5 to 9 or 10 or more prescribed medications (Figure; eTables 3 and 4 in Supplement 1). However, people with lower education were less likely to have HFRS higher than 0 when receiving a dementia diagnosis (OR, 0.87; 95% CI, 0.84-0.91 and OR, 0.82; 95% CI, 0.78-0.86, respectively).

**Figure.**
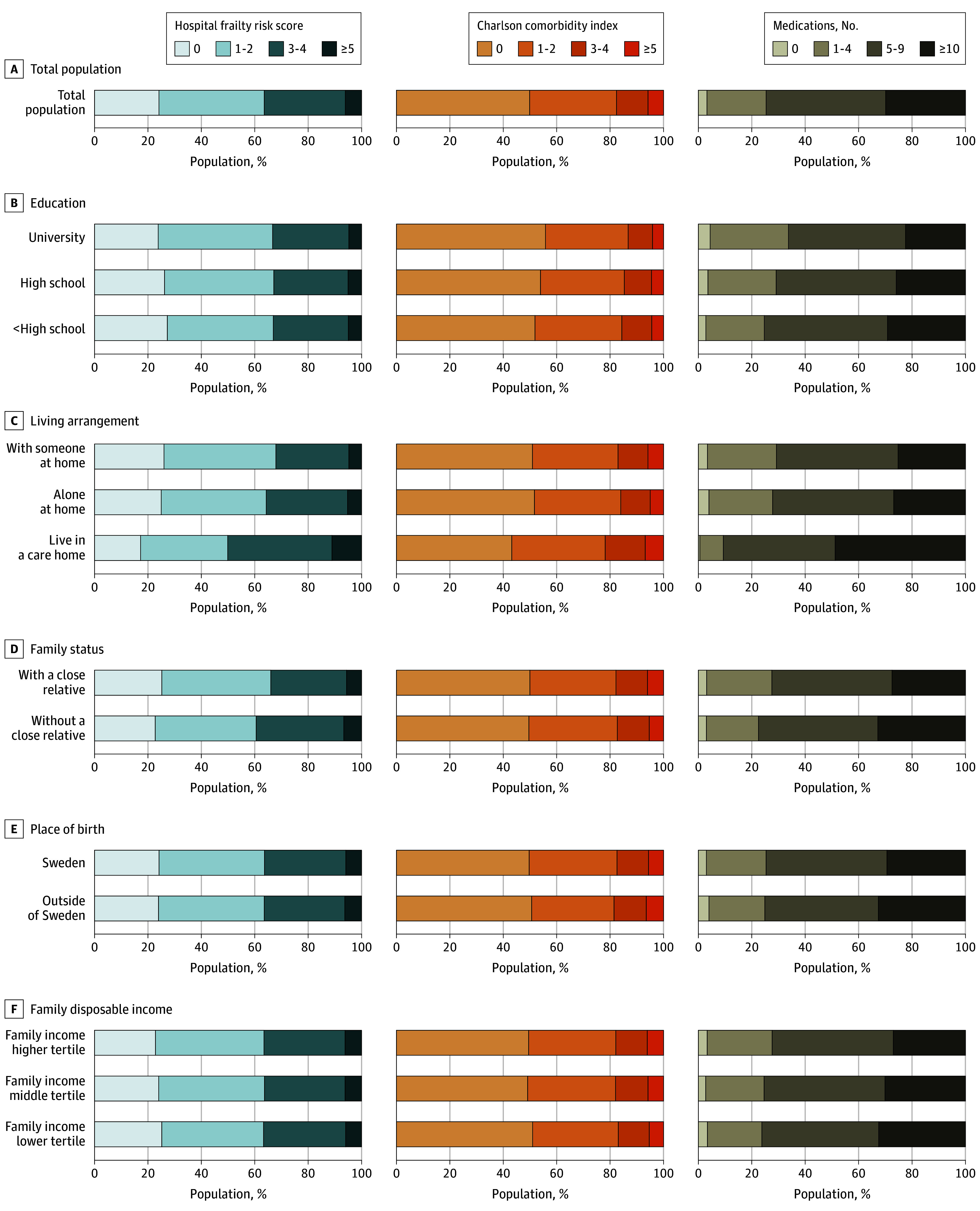
Hospital Frailty Risk Score, Charlson Comorbidity Index, and Number of Medications at Time of Dementia Diagnosis in the Total Population and Stratified by Education, Living Arrangements, Family Status, Place of Birth, and Family Disposable Income

Individuals who were living alone at home or in a care home when diagnosed with dementia were more likely to have an HFRS higher than 0 and CCI of 1 or more than cohabiting individuals, and the association was greater for care home residents (OR, 1.56; 95% CI, 1.54-1.59 and OR, 1.20; 95% CI, 1.19-1.22, respectively) (Table 2). Moreover, at dementia diagnosis, individuals living alone and care home residents had a higher proportion of HFRS categories 5 to 14 and 15 or higher (Figure; eTable 5 in Supplement 1) compared with cohabitating individuals. Care home residents diagnosed with dementia also had a higher share of CCI categories 1 to 2, 3 to 4, and 5 or more, and 5 to 9 or 10 or more prescribed medications than cohabiting individuals (Figure; eTables 3 and 4 in Supplement 1). Similar patterns were found for family status, where people without a close relative at dementia diagnosis had higher HFRS, higher CCI, and more prescribed medications compared with those with a close relative (Table 2 and Figure; eTables 3 and 5 in Supplement 1).

The distribution of HFRS did not differ substantially by place of birth. Individuals in the lower family income tertile were slightly less likely to have an HFRS greater than 0 at dementia diagnosis (Table 2). Individuals born outside of Sweden or those with lower family income at dementia diagnosis were more likely to have a CCI of 1 or higher and 5 or more prescribed medications compared with Swedish born individuals and those with higher family income (Table 2); they were also more likely to have CCI categories 3 to 4 and 5 or more at the time of dementia diagnosis (eTable 3 in Supplement 1).

The association between sociodemographic factors and age and health status at the time of dementia diagnosis was similar among men and women (eTable 6 in Supplement 1). When stratified by rural or urban areas, the association of living alone and being born outside of Sweden with health status at dementia diagnosis was attenuated in rural areas (eTable 7 in Supplement 1). Restricting the study population to those identified by specialist care diagnosis alone revealed similar results in terms of age and sociodemographic factors, but the association for people without a close relative and people living alone in relation to CCI and medications became statistically insignificant (eTable 8 in Supplement 1). Finally, results remained largely unchanged when restricting the study period to pre–COVID-19 (eTable 9 in Supplement 1).

## Discussion

In this nationwide study, individuals living alone at home, in a care home, without a close relative, or with low education were a mean of 3.1 years older when diagnosed with dementia compared with their counterparts. They were also frailer and had more comorbidities and prescriptions at the time of dementia diagnosis despite accounting for age differences, which may indicate a more advanced disease and/or a poorer prognosis for these groups.

Etiological studies have consistently showed social isolation and lower socioeconomic status to be associated with higher risk and younger onset of dementia.^11,12,30,31,32^ We, on the other hand, report older ages at diagnosis for these subgroups, which suggests a delayed diagnosis. It is likely that, even though the underlying risk of dementia is elevated in these vulnerable subgroups, prompting an earlier onset, delays in diagnostic investigation for dementia result in a formal clinical diagnosis at older ages. Past screening cohorts have reported 2 to 3 years as the mean duration from dementia symptom onset to a clinical diagnosis.^17,18,33,34^ Living alone has been associated with a lower likelihood of receiving dementia diagnostic investigations.^14^ The results for education and timing of dementia diagnosis have been mixed, with some studies suggesting a greater delay in lower-educated individuals^19,35^ and others reporting the opposite.^17,20^ However, our results are not directly comparable with these previous findings as we do not focus on determinants of diagnostic delays and cannot compare individuals with undetected dementia. Nevertheless, qualitative and mixed method studies have identified low education, lack of knowledge of dementia, lack of informal support, and living arrangement as important patient-related barriers to a timely dementia diagnosis,^36^ all of which support our results. Moreover, delayed diagnosis could also relate to potential negative consequences of a dementia diagnosis. For instance, a US study showed that dementia diagnosis is documented later in states that require dementia diagnosis to be reported to motor vehicles agencies.^37^ Therefore, risk of losing a driving license could be part of an explanation for our findings, particularly among people who live alone or without a close relative and are dependent on driving a car. At the same time, these people might need special housing or institutionalization the most, which, on the other hand, might speed up the time to diagnosis.

Dementia impacts both cognition and physical health. A recent study^22^ using Medicare claims data reported frailty index, which is highly correlated with functional limitations,^38^ as a useful proxy for dementia severity. Higher comorbidity burden is also correlated with a more advanced stage of dementia.^23,39^ This suggests that disadvantaged subgroups in our study could be at a more advanced stage of dementia when diagnosed, given their higher burden of frailty, comorbidity, and number of dispensed drugs. At the same time, dementia also adversely affects and complicates the care of coexisting conditions, undermines individuals’ ability to self-manage their health, and affects utilization of care.^40,41,42^ Therefore, disadvantaged subgroups may face a poorer prognosis of both dementia and coexisting conditions. In our study, it is perhaps not surprising that care home residents were much older and in worse health when diagnosed with dementia, as oftentimes a formal dementia diagnosis is not needed anymore to receive adequate care. However, for other subgroups, such as those living alone or without a close relative, efforts are needed to foster collaborations between health services and municipalities to work proactively with these groups for a timely diagnosis of dementia.

### Limitations

This study had limitations. Our study focused on individuals receiving a dementia diagnosis, not the total population at risk, and therefore does not allow interpretation of risk factors for dementia nor estimates of diagnostic delays. The focus was to describe how subgroups differ at dementia diagnosis, which could infer a diagnostic delay and/or impact prognosis. We did not have access to dementia diagnosed in primary care alone and not dispensed any drugs, leading to some missed cases. Although it has been shown that 95% of dementia diagnoses in Stockholm could be captured by specialist care or antidementia drug use,^8^ it is not known to what extent this is generalizable at the national level; the proportion of missed cases could be higher in rural areas. This could result in an underestimated association between sociodemographic factors and worse health at dementia diagnosis, since past data suggest that patients with dementia attending primary care are older, more likely to reside in a care home, and have more comorbidities than those attending specialist care.^43^ Indeed, the association for some subgroups (eg, living alone) was attenuated when restricting to dementia diagnosed in specialist care or in rural areas, suggesting that these groups may have used primary care to a larger extent for both dementia and coexisting conditions. Another issue is that cases identified through drug prescriptions could have been diagnosed earlier in primary care, which could result in an overestimated association if vulnerable groups are more often diagnosed in primary care only and prescribed drugs later. We believe this is unlikely, however, since drug treatment would be adopted very close to the diagnosis, as there is no point in delaying the prescription.

Missed primary care diagnoses may also affect the assessment of CCI and HFRS. Past evidence suggests lower socioeconomic subgroups were more likely to visit primary care than specialist care.^44^ This would result in an underestimation of the comorbidity and frailty level among the vulnerable subgroups in this study, particularly in rural areas. This may also explain why individuals with lower education or lower family income were less likely to have an HFRS greater than 0 at dementia diagnosis. Yet most subgroups were more likely to have 5 or more medications, as prescriptions come from both primary and specialist care. Moreover, as current antidementia drugs are mostly used to treat AD and mixed dementia, other dementia types (eg, vascular dementia) might be missed by drug prescriptions.

## Conclusions

In Sweden, among individuals with a new dementia diagnosis identified through specialist care or antidementia drugs, individuals living alone, in a care home, without a close relative, or with low education were a mean of 3.1 years older when diagnosed with dementia compared with cohabiting individuals and individuals with a close relative or with higher education. These individuals were also frailer, had more comorbidities, and were taking more medications at the time of diagnosis, even after adjusting for age differences. This suggests greater delays in dementia diagnosis within these subgroups and a poorer prognosis for both dementia and coexisting conditions. Future efforts should clarify how health services can be improved to meet the needs of dementia diagnosis and care among these vulnerable subgroups.

## Notes

Supplement 1. **eTable 1. **Swedish ICD Codes Used to Create the Charlson Comorbidity Index **eTable 2.** Swedish ICD 10 Codes Used to Create the Hospital Frailty Risk Score **eTable 3.** Relative Risk Ratio and 95% CI of Charlson Comorbidity Index Categories Compared With 0 by Sociodemographic Factors **eTable 4.** Relative Risk Ratio and 95% CI of Number of Prescribed Medications Compared With 0, by Sociodemographic Factors **eTable 5.** Relative Risk Ratio and 95% CI of Hospital Frailty Risk Score Categories Compared With 0, by Sociodemographic Factors **eTable 6. **Health Status at the Time of Dementia Diagnosis Stratified by Sociodemographic Factors and Sex **eTable 7.** Health Status at the Time of Dementia Diagnosis Stratified by Sociodemographic Factors and Rural/Urban Areas **eTable 8.** Age and Health Status at the Time of Dementia Diagnosis Stratified by Sociodemographic Factors Among Individuals Identified by Specialist Care Diagnosis Alone (n=48,295) **eTable 9.** Age and Health Status at the Time of Dementia Diagnosis Stratified by Sociodemographic Factors Among Individuals Identified Between January 1, 2018 and December 31, 2019 (n=43878)

Supplement 2.
Data Sharing Statement
