# Supplementary material for: Social Factors, Age, and Health at Time of Dementia Diagnosis
Source: JAMA Netw Open. 2025 Feb 21;8(2):e2461117. doi: 10.1001/jamanetworkopen.2024.61117 (PMC11846011; doi:10.1001/jamanetworkopen.2024.61117)
Supplement: Supplement 1. — eTable 1. Swedish ICD Codes Used to Create the Charlson Comorbidity Index eTable 2. Swedish ICD 10 Codes Used to Create the Hospital Frailty Risk Score eTable 3. Relative Risk Ratio and 95% CI of Charlson Comorbidity Index Categories Compared With 0 by Sociodemographic Factors eTable 4. Relative Risk Ratio and 95% CI of Number of Prescribed Medications Compared With 0, by Sociodemographic Factors eTable 5. Relative Risk Ratio and 95% CI of Hospital Frailty Risk Score Categories Compared With 0, by Sociodemographic Factors eTable 6. Health Status at the Time of Dementia Diagnosis Stratified by Sociodemographic Factors and Sex eTable 7. Health Status at the Time of Dementia Diagnosis Stratified by Sociodemographic Factors and Rural/Urban Areas eTable 8. Age and Health Status at the Time of Dementia Diagnosis Stratified by Sociodemographic Factors Among Individuals Identified by Specialist Care Diagnosis Alone (n=48,295) eTable 9. Age and Health Status at the Time of Dementia Diagnosis Stratified by Sociodemographic Factors Among Individuals Identified Between January 1, 2018 and December 31, 2019 (n=43878) [file jamanetwopen-e2461117-s001.pdf]

Supplemental Online Content

Ding M, Schmidt-Mende K, Modig K. Social factors, age, and health at time of dementia diagnosis. *JAMA Netw Open*. 2025;8(2):e2461117.
doi:10.1001/jamanetworkopen.2024.61117

eTable 1. Swedish ICD Codes Used to Create the Charlson Comorbidity Index

eTable 2. Swedish ICD 10 Codes Used to Create the Hospital Frailty Risk Score

eTable 3. Relative Risk Ratio and 95% CI of Charlson Comorbidity Index Categories Compared With 0 by Sociodemographic Factors

eTable 4. Relative Risk Ratio and 95% CI of Number of Prescribed Medications Compared With 0, by Sociodemographic Factors

eTable 5. Relative Risk Ratio and 95% CI of Hospital Frailty Risk Score Categories Compared With 0, by Sociodemographic Factors

eTable 6. Health Status at the Time of Dementia Diagnosis Stratified by Sociodemographic Factors and Sex

eTable 7. Health Status at the Time of Dementia Diagnosis Stratified by Sociodemographic Factors and Rural/Urban Areas

eTable 8. Age and Health Status at the Time of Dementia Diagnosis Stratified by Sociodemographic Factors Among Individuals Identified by Specialist Care Diagnosis Alone (n=48,295)

eTable 9. Age and Health Status at the Time of Dementia Diagnosis Stratified by Sociodemographic Factors Among Individuals Identified Between January 1, 2018 and December 31, 2019 (n=43878)

This supplemental material has been provided by the authors to give readers additional information about their work.

© 2025 Ding M et al. *JAMA Network Open*.

eTable 1. Swedish ICD codes used to create the Charlson Comorbidity Index

ICD 10	Disease	Score
I22-I23, I252	Myocardial infarction	1
I11, I13, I255, I42–43, I50, I517	Congestive heart failure	1
I70–73, I770-I771, K551, K558–559, R02, Z958–959	Peripheral vascular disease	1
G45–46, I60–69	Cerebrovascular disease	1
A810, F00–03, F051, G30–31	Dementia	1
I26–27, J40-J47, J60–67, J684, J701, J703	Chronic pulmonary disease	1
M05–06, M09, M120, M315, M32-M36	Rheumatic disease	1
B18, I85, I864, I982, K70–71, K721, K729, K76, R162, Z944	Liver disease	2
E10–14	Diabetes mellitus	2
G114, G81–83	Hemiplegia/paraplegia	2
I12–13, N01, N03, N05, N07, N08, N171, N172, N18, N19, N25, Z49, Z940, Z992	Renal disease	2
C00–26, C30–34, C37–41, C43, C45–58, C60–76, C80–85, C88, C90–97	Malignancy	2
C77–79	Metastatic tumours	6
B20–24	AIDS/HIV	6

© 2025 Ding M et al. *JAMA Network Open*.

eTable 2. Swedish ICD 10 codes used to create the Hospital Frailty Risk Score

ICD10	ICD description	Weight
F00	Dementia in Alzheimer's disease	7.1
G81	Hemiplegia	4.4
G30	Alzheimer's disease	4.0
I69	Sequelae of cerebrovascular disease	3.7
R29	Other symptoms and signs involving the nervous and musculoskeletal systems	3.6
N39	Other disorders of urinary system (includes urinary tract infection and urinary incontinence)	3.2
F05	Delirium, not induced by alcohol and other psychoactive substances	3.2
W19	Unspecified fall	3.2
S00	Superficial injury of head	3.2
R31	Unspecified haematuria	3.0
B96	Other bacterial agents as the cause of diseases classified to other chapters	2.9
R41	Other symptoms and signs involving cognitive functions and awareness	2.7
R26	Abnormalities of gait and mobility	2.6
I67	Other cerebrovascular diseases	2.6
R56	Convulsions, not elsewhere classified	2.6
R40	Somnolence, stupor and coma	2.5
T83	Complications of genitourinary prosthetic devices, implants and grafts	2.4
S06	Intracranial injury	2.4
S42	Fracture of shoulder and upper arm	2.3
E87	Other disorders of fluid, electrolyte and acid-base balance	2.3
M25	Other joint disorders, not elsewhere classified	2.3
E86	Volume depletion	2.3
R54	Senility	2.2
Z51	Care involving use of rehabilitation procedures	2.1
F03	Unspecified dementia	2.1
W18	Other fall on same level	2.1
Z75	Problems related to medical facilities and other health care	2.0
F01	Vascular dementia	2.0
S80	Superficial injury of lower leg	2.0
L03	Cellulitis	2.0
H54	Blindness and low vision	1.9
E53	Deficiency of other B group vitamins	1.9
Z60	Problems related to social environment	1.8
G20	Parkinson's disease	1.8
R55	Syncope and collapse	1.8
S22	Fracture of rib(s), sternum and thoracic spine	1.8
K59	Other functional intestinal disorders	1.8
N17	Acute renal failure	1.8
L89	Decubitus ulcer	1.7
Z22	Carrier of infectious disease	1.7
B95	Streptococcus and staphylococcus as the cause of diseases classified to other chapters	1.7
L97	Ulcer of lower limb, not elsewhere classified	1.6
R44	Other symptoms and signs involving general sensations and perceptions	1.6

© 2025 Ding M et al. *JAMA Network Open*.

K26	Duodenal ulcer	1.6
I95	Hypotension	1.6
N19	Unspecified renal failure	1.6
A41.9	Other septicaemia	1.6
Z87	Personal history of other diseases and conditions	1.5
J96	Respiratory failure, not elsewhere classified	1.5
X59	Exposure to unspecified factor	1.5
M19	Other arthrosis	1.5
G40	Epilepsy	1.5
M81	Osteoporosis without pathological fracture	1.4
S72	Fracture of femur	1.4
S32	Fracture of lumbar spine and pelvis	1.4
E16	Other disorders of pancreatic internal secretion	1.4
R94	Abnormal results of function studies	1.4
N18	Chronic renal failure	1.4
R33	Retention of urine	1.3
R69	Unknown and unspecified causes of morbidity	1.3
N28	Other disorders of kidney and ureter, not elsewhere classified	1.3
R32	Unspecified urinary incontinence	1.2
G31	Other degenerative diseases of nervous system, not elsewhere classified	1.2
Y95	Nosocomial condition	1.2
S09	Other and unspecified injuries of head	1.2
R45	Symptoms and signs involving emotional state	1.2
G45	Transient cerebral ischaemic attacks and related syndromes	1.2
Z74	Problems related to care-provider dependency	1.1
M79	Other soft tissue disorders, not elsewhere classified	1.1
W06	Fall involving bed	1.1
S01	Open wound of head	1.1
A04	Other bacterial intestinal infections	1.1
A09	Diarrhoea and gastroenteritis of presumed infectious origin	1.1
J18	Pneumonia, organism unspecified	1.1
J69	Pneumonitis due to solids and liquids	1.0
R47	Speech disturbances, not elsewhere classified	1.0
E55	Vitamin D deficiency	1.0
Z93	Artificial opening status	1.0
R02	Gangrene, not elsewhere classified	1.0
R63	Symptoms and signs concerning food and fluid intake	0.9
H91	Other hearing loss	0.9
W10	Fall on and from stairs and steps	0.9
W01	Fall on same level from slipping, tripping and stumbling	0.9
E05	Thyrotoxicosis [hyperthyroidism]	0.9
M41	Scoliosis	0.9
R13	Dysphagia	0.8
Z99	Dependence on enabling machines and devices	0.8
U82.0	Agent resistant to penicillin and related antibiotics	0.8
M80	Osteoporosis with pathological fracture	0.8
K92	Other diseases of digestive system	0.8
I63	Cerebral Infarction	0.8
N20	Calculus of kidney and ureter	0.7

© 2025 Ding M et al. *JAMA Network Open*.

F10	Mental and behavioural disorders due to use of alcohol	0.7
Y84	Other medical procedures as the cause of abnormal reaction of the patient	0.7
R00	Abnormalities of heart beat	0.7
J22	Unspecified acute lower respiratory infection	0.7
Z73	Problems related to life-management difficulty	0.6
R79	Other abnormal findings of blood chemistry	0.6
Z91	Personal history of risk-factors, not elsewhere classified	0.5
S51	Open wound of forearm	0.5
F32	Depressive episode	0.5
M48.0	Spinal stenosis (secondary code only)	0.5
E83	Disorders of mineral metabolism	0.4
M15	Polyarthrosis	0.4
D64	Other anaemias	0.4
L08	Other local infections of skin and subcutaneous tissue	0.4
R11	Nausea and vomiting	0.3
K52	Other noninfective gastroenteritis and colitis	0.3
R50	Fever of unknown origin	0.1

© 2025 Ding M et al. *JAMA Network Open*.

eTable 3. Relative risk ratio and 95% confidence interval of Charlson Comorbidity Index categories compared to zero, by sociodemographic factors.

Socio-demographic factors	Charlson Comorbidity Index categories vs 0		
	1-2	3-4	≥5
Education level			
University	Ref (1.00)	Ref (1.00)	Ref (1.00)
High school	1.04 (1.00-1.08)[b]	1.15 (1.07-1.22)[a]	1.13 (1.03-1.23)[b]
Lower than high school	1.06 (1.01-1.20)[b]	1.25 (1.17-1.34)[a]	1.13 (1.02-1.24)[b]
Living arrangement			
Live with someone at home	Ref (1.00)	Ref (1.00)	Ref (1.00)
Live alone at home	1.03 (1.00-1.07)[a]	1.10 (1.05-1.15)[a]	1.05 (0.99-1.12)
Live in a care home	1.20 (1.15-1.25)[a]	1.53 (1.44-1.61)[a]	1.52 (141-1.64)[a]
Family status			
With a close relative	Ref (1.00)	Ref (1.00)	Ref (1.00)
Without a close relative	1.05 (1.02-1.08)[a]	1.11 (1.07-1.16)[a]	1.05 (0.99-1.11)[b]
Place of birth			
Born in Sweden	Ref (1.00)	Ref (1.00)	Ref (1.00)
Born outside of Sweden	0.98 (0.94-1.04)	1.08 (1.02-1.15)[b]	1.19 (1.10-1.28)[a]
Family disposable income			
Higher tertile	Ref (1.00)	Ref (1.00)	Ref (1.00)
Middle tertile	1.02 (0.97-1.09)	1.09 (1.04-1.15)[a]	1.02 (0.92-1.13)
Lower tertile	1.01 (0.96-1.07)	1.15 (1.09-1.21)[a]	1.09 (0.98-1.21)

Relative risk ratios are from multinomial logistic regressions and adjusted for age at diagnosis and sex. [a]p<0.001; [b]p<0.05

© 2025 Ding M et al. *JAMA Network Open*.

eTable 4. Relative risk ratio and 95% confidence interval of number of prescribed medications compared to zero, by sociodemographic factors.

Socio-demographic factors	Number of prescribed medications vs 0		
	1-4	5-9	≥10
Education level			
University	Ref (1.00)	Ref (1.00)	Ref (1.00)
High school	1.09 (0.97-1.20)	1.38 (1.24-1.53)[a]	1.68 (1.51-1.87)[a]
Lower than high school	1.08 (0.97-1.19)	1.24 (1.13-1.37)[a]	1.41 (1.27-1.55)[a]
Living arrangement			
Live with someone at home	Ref (1.00)	Ref (1.00)	Ref (1.00)
Live alone at home	0.73 (0.67-1.78)[a]	0.72 (0.67-0.78)[a]	0.75 (0.69-0.81)[a]
Live in a care home	1.80 (1.46-2.21)[a]	3.74 (3.06-4.58)[a]	7.30 (5.96-8.94)[a]
Family status			
With a close relative	Ref (1.00)	Ref (1.00)	Ref (1.00)
Without a close relative	0.80 (0.74-0.87)[a]	0.89 (0.82-0.96)[b]	1.05 (0.99-1.11)
Place of birth			
Born in Sweden	Ref (1.00)	Ref (1.00)	Ref (1.00)
Born outside of Sweden	0.73 (0.65-0.80)[a]	0.77 (0.70-0.85)[a]	0.91 (0.82-1.00)
Family disposable income			
Higher tertile	Ref (1.00)	Ref (1.00)	Ref (1.00)
Middle tertile	1.07 (0.92-1.23)	1.18 (1.08-1.29)[a]	1.31 (1.19-1.43)[b]
Lower tertile	0.81 (0.70-0.93)[b]	0.95 (0.82-1.09)	1.17 (1.07-1.27)[a]

Relative risk ratios are from multinomial logistic regressions and adjusted for age at diagnosis and sex. [a]$p<0.001$; [b]$p<0.05$

© 2025 Ding M et al. *JAMA Network Open*.

eTable 5. Relative risk ratio and 95% confidence interval of Hospital Frailty Risk Score categories compared to zero, by sociodemographic factors.

**Socio-demographic factors**	**Hospital Frailty Risk Score categories vs 0**		
	1-4	5-14	≥15
Education level			
University	Ref (1.00)	Ref (1.00)	Ref (1.00)
High school	0.84 (0.80-0.88)[a]	0.87 (0.82-0.92)[a]	0.95 (0.80-0.96)[a]
Lower than high school	0.81 (0.77-0.85)[a]	0.83 (0.79-0.87)[a]	0.88 (0.79-0.96)[a]
Living arrangement			
Live with someone at home	Ref (1.00)	Ref (1.00)	Ref (1.00)
Live alone at home	1.02 (1.01-1.08)[b]	1.23 (1.18-1.28)[a]	1.36 (1.27-1.45)[a]
Live in a care home	1.23 (1.17-1.30)[a]	2.17 (2.06-2.29)[a]	3.98 (3.69-4.29)[a]
Family status			
With a close relative	Ref (1.00)	Ref (1.00)	Ref (1.00)
Without a close relative	1.07 (1.04-1.11)[a]	1.25 (1.20-1.30)[a]	1.35 (1.29-1.42)[a]
Place of birth			
Born in Sweden	Ref (1.00)	Ref (1.00)	Ref (1.00)
Born outside of Sweden	1.00 (0.95-1.04)	1.00 (0.95-1.05)	1.10 (1.01-1.19)[b]
Family disposable income			
Higher tertile	Ref (1.00)	Ref (1.00)	Ref (1.00)
Middle tertile	0.93 (0.90-0.97)[b]	0.95 (0.92-0.99)[b]	0.97 (0.91-1.04)
Lower tertile	0.87 (0.83-0.90)[a]	0.96 (0.92-1.00)[b]	1.00 (0.94-1.08)

Relative risk ratios are from multinomial logistic regressions and adjusted for age at diagnosis and sex. [a]$p<0.001$; [b]$p<0.05$

© 2025 Ding M et al. *JAMA Network Open*.

eTable 6. Health status at the time of dementia diagnosis stratified by sociodemographic factors and sex

Socio-demographic factors	Age at diagnosis, mean (SD)		OR (95% CI) of HFRS >0		OR (95% CI) of CCI ≥1		OR (95% CI) of medication ≥5	
	Men	Women	Men	Women	Men	Women	Men	Women
Education level								
University	79.1 (7.3)	80.0 (7.5)	Ref (1.00)	Ref (1.00)	Ref (1.00)	Ref (1.00)	Ref (1.00)	Ref (1.00)
High school	79.1 (7.3)	80.8 (7.5)	0.82 (0.77-0.88)[a]	0.88 (0.83-0.93)[a]	1.06 (1.00-1.12)[b]	1.09 (1.03-1.14)[b]	1.18 (1.11-1.25)[a]	1.25 (1.18-1.53)[a]
Lower than high school	81.0 (7.1)	83.9 (7.2)	0.79 (0.74-0.85)[a]	0.80 (0.76-0.84)[a]	1.09 (1.03-1.16)[b]	1.13 (1.08-1.19)[a]	1.30 (1.22-1.38)[a]	1.25 (1.18-1.32)[a]
Living arrangement								
Live with someone at home	80.2 (7.0)	79.5 (7.1)	Ref (1.00)	Ref (1.00)	Ref (1.00)	Ref (1.00)	Ref (1.00)	Ref (1.00)
Live alone at home	81.0 (7.9)	83.7 (7.2)	1.13 (1.07-1.19)[a]	1.13 (1.09-1.17)[a]	1.01 (0.96-1.05)	1.10 (1.05-1.14)[a]	0.89 (0.84-0.93)[a]	1.05 (1.01-1.09)[b]
Live in a care home	84.6 (7.6)	87.8 (6.8)	1.75 (1.63-1.88)[a]	1.64 (1.55-1.73)[a]	1.17 (1.10-1.24)[a]	1.26 (1.19-1.32)[a]	2.73 (2.51-2.98)[a]	3.14 (2.92-3.38)[a]
Family status								
With a close relative	80.7 (7.2)	80.2 (7.2)	Ref (1.00)	Ref (1.00)	Ref (1.00)	Ref (1.00)	Ref (1.00)	Ref (1.00)
Without a close relative	81.8 (8.0)	85.1 (7.3)	1.19 (1.14-1.25)[a]	1.15 (1.11-1.20)[a]	1.03 (0.99-1.07)	1.11 (1.07-1.15)[a]	1.03 (0.98-1.08)	1.13 (1.09-1.18)[a]
Place of birth								
Born in Sweden	81.2 (7.5)	83.2 (7.7)	Ref (1.00)	Ref (1.00)	Ref (1.00)	Ref (1.00)	Ref (1.00)	Ref (1.00)
Born outside of Sweden	79.8 (7.6)	82.0 (7.8)	0.99 (0.93-1.06)	1.01 (0.96-1.07)	1.01 (0.95-1.07)	0.97 (0.93-1.02)	1.05 (0.98-1.11)	1.05 (0.99-1.11)
Family disposable income								
Higher tertile	81.4 (7.5)	82.3 (7.9)	Ref (1.00)	Ref (1.00)	Ref (1.00)	Ref (1.00)	Ref (1.00)	Ref (1.00)
Middle tertile	81.3 (7.1)	83.0 (7.7)	0.91 (0.86-0.95)[a]	0.97 (0.93-1.02)	1.02 (0.98-1.07)	1.10 (1.05-1.14)[a]	1.11 (1.06-1.16)[a]	1.21 (1.15-1.27)[a]
Lower tertile	79.9 (7.9)	83.3 (7.5)	0.95 (0.90-1.01)	0.89 (0.85-0.93)[a]	1.06 (1.01-1.11)[b]	1.09 (1.05-1.13)[a]	1.19 (1.12-1.25)[a]	1.27 (1.22-1.33)[a]

ORs are adjusted for age at diagnosis. [a]p<0.001; [b]p<0.05

© 2025 Ding M et al. *JAMA Network Open*.

eTable 7. Health status at the time of dementia diagnosis stratified by sociodemographic factors and rural/urban areas.

Socio-demographic factors	Age at diagnosis, mean (SD)		OR (95% CI) of HFRS >0		OR (95% CI) of CCI ≥1		OR (95% CI) of medication ≥5	
	Rural (n=25,903)	Urban (n=81,804)	Rural (n=25,903)	Urban (n=81,804)	Rural (n=25,903)	Urban (n=81,804)	Rural (n=25,903)	Urban (n=81,804)
Education level								
University	79.3 (7.3)	79.7 (7.4)	Ref (1.00)	Ref (1.00)	Ref (1.00)	Ref (1.00)	Ref (1.00)	Ref (1.00)
High school	79.4 (7.5)	80.3 (7.5)	0.91 (0.82-0.99)[b]	0.88 (0.84-0.93)[a]	1.10 (1.01-1.20)[b]	1.08 (1.03-1.12)[b]	1.33 (1.21-1.45)[a]	1.19 (1.14-1.24)[a]
Lower than high school	82.5 (7.1)	82.8 (7.3)	0.90 (0.82-0.99)[b]	0.84 (0.79-0.88)[a]	1.13 (1.04-1.23)[b]	1.12 (1.08-1.17)[a]	1.40 (1.33-1.47)[a]	1.39 (1.33-1.46)[a]
Living arrangement								
Live with someone at home	79.7 (7.0)	79.9 (7.1)	Ref (1.00)	Ref (1.00)	Ref (1.00)	Ref (1.00)	Ref (1.00)	Ref (1.00)
Live alone at home	82.8 (7.4)	82.9 (7.5)	1.00 (0.94-1.06)	1.17 (1.12-1.21)[a]	0.98 (0.92-1.03)	1.07 (1.04-1.11)[a]	0.99 (0.96-1.03)	0.97 (1.91-1.03)
Live in a care home	86.8 (7.2)	86.6 (7.3)	1.71 (1.56-1.87)[a]	1.77 (1.67-1.86)[a]	1.28 (1.19-1.38)[a]	1.31 (1.26-1.37)[a]	2.98 (2.66-3.33)[a]	3.27 (3.07-3.48)[a]
Family status								
With a close relative	80.3 (7.2)	80.6 (7.2)	Ref (1.00)	Ref (1.00)	Ref (1.00)	Ref (1.00)	Ref (1.00)	Ref (1.00)
Without a close relative	84.1 (7.6)	84.1 (7.7)	1.11 (1.05-1.18)[a]	1.21 (1.17-1.25)[a]	1.03 (0.98-1.09)	1.07 (1.04-1.11)[a]	1.15 (1.11-1.19)[a]	1.14 (1.10-1.18)[a]
Place of birth								
Born in Sweden	82.0 (7.6)	82.4 (7.7)	Ref (1.00)	Ref (1.00)	Ref (1.00)	Ref (1.00)	Ref (1.00)	Ref (1.00)
Born outside of Sweden	80.7 (7.6)	81.2 (7.8)	0.86 (0.78-0.94)[a]	1.04 (0.99-1.09)	0.92 (0.83-1.00)	1.03 (0.99-1.07)	0.89 (0.81-0.98)[b]	1.14 (1.09-1.20)[a]
Family disposable income								
Higher tertile	81.1 (7.6)	82.0 (7.7)	Ref (1.00)	Ref (1.00)	Ref (1.00)	Ref (1.00)	Ref (1.00)	Ref (1.00)
Middle tertile	81.8 (7.5)	82.4 (7.5)	1.11 (1.03-1.19)[b]	0.92 (0.88-0.95)[a]	1.12 (1.04-1.19)[b]	1.05 (1.03-1.10)[a]	1.34 (1.24-1.44)[a]	1.11 (1.07-1.15)[a]
Lower tertile	82.7 (7.7)	82.2 (7.8)	1.06 (0.98-1.13)	0.90 (0.86-0.94)[b]	1.13 (1.06-1.19)[a]	1.06 (1.02-1.10)[a]	1.35 (1.25-1.45)[a]	1.20 (1.15-1.25)[a]

ORs are adjusted for age and sex at diagnosis. [a]p<0.001; [b]p<0.05

© 2025 Ding M et al. *JAMA Network Open*.

eTable 8. Age and health status at the time of dementia diagnosis stratified by sociodemographic factors among individuals identified by specialist care diagnosis alone (n=48,295).

**Socio-demographic factors**	Age at diagnosis, mean (SD)	OR (95% CI) of HFRS >0	OR (95% CI) of CCI ≥1	OR (95% CI) of medications ≥5
Education level				
University	80.7 (8.0)	Ref (1.00)	Ref (1.00)	Ref (1.00)
High school	80.9 (8.3)	0.89 (0.82-0.97)[a]	1.10 (1.03-1.17)[a]	1.22 (1.17-1.27)[a]
Lower than high school	83.7 (7.8)	0.80 (0.73-0.86)[a]	1.11 (1.04-1.19)[a]	1.37 (1.28-1.48)[a]
Living arrangement				
Live with someone at home	81.0 (7.7)	Ref (1.00)	Ref (1.00)	Ref (1.00)
Live alone at home	83.7 (8.0)	0.99 (0.94-1.05)	0.96 (0.92-1.01)	0.84 (0.80-0.88)
Live in a care home	86.7 (7.4)	1.15 (1.08-1.23)[a]	1.09 (1.04-1.15)[a]	2.81 (2.60-3.03)[a]
Family status				
With a close relative	81.8 (7.7)	Ref (1.00)	Ref (1.00)	Ref (1.00)
Without a close relative	85.0 (8.0)	1.00 (0.95-1.05)	0.97 (0.93-1.01)	1.01 (0.96-1.06)
Place of birth				
Born in Sweden	83.7 (8.0)	Ref (1.00)	Ref (1.00)	Ref (1.00)
Born outside of Sweden	82.2 (8.1)	0.97 (0.91-1.04)	0.97 (0.92-1.02)	0.98 (0.92-1.05)
Family disposable income				
Higher tertile	83.8 (7.9)	Ref (1.00)	Ref (1.00)	Ref (1.00)
Middle tertile	83.7 (7.8)	0.88 (0.84-0.96)[a]	0.99 (0.95-1.04)	1.07 (1.01-1.13)[b]
Lower tertile	83.1 (8.3)	0.76 (0.71-0.81)[a]	0.92 (0.88-0.97)[a]	1.03 (0.98-1.08)

ORs are adjusted for age at diagnosis and sex. HFRS= Hospital Frailty Risk Score; CCI= Charlson Comorbidity Index; CI=confidence interval; OR=odds ratio; SD=standard deviation. [a]p<0.001; [b]p<0.05.

© 2025 Ding M et al. *JAMA Network Open*.

eTable 9. Age and health status at the time of dementia diagnosis stratified by sociodemographic factors among individuals identified between 2018 January 1 and 2019 December 31 (n=43878).

**Socio-demographic factors**	Age at diagnosis, mean (SD)	OR (95% CI) of HFRS >0	OR (95% CI) of CCI ≥1	OR (95% CI) of medications ≥5
Education level				
University	78.1 (7.4)	Ref (1.00)	Ref (1.00)	Ref (1.00)
High school	78.6 (7.5)	0.84 (0.78-0.91)[a]	1.09 (1.02-1.16)[b]	1.21 (1.13-1.30)[a]
Lower than high school	81.1 (7.2)	0.75 (0.69-0.82)[a]	1.08 (1.00-1.16)[b]	1.34 (1.24-1.44)[a]
Living arrangement				
Live with someone at home	79.6 (7.3)	Ref (1.00)	Ref (1.00)	Ref (1.00)
Live alone at home	82.7 (7.7)	1.10 (1.05-1.16)[a]	1.05 (1.00-1.09)[b]	0.97 (0.93-1.02)
Live in a care home	86.7 (7.3)	1.53 (1.43-1.65)[a]	1.30 (1.23-1.37)[a]	3.02 (2.78-3.28)[a]
Family status				
With a close relative	80.1 (7.4)	Ref (1.00)	Ref (1.00)	Ref (1.00)
Without a close relative	84.0 (7.7)	1.16 (1.10-1.21)[a]	1.06 (1.02-1.11)[b]	1.15 (1.10-1.21)[a]
Place of birth				
Born in Sweden	82.2 (7.8)	Ref (1.00)	Ref (1.00)	Ref (1.00)
Born outside of Sweden	80.7 (8.0)	1.07 (1.00-1.15)	1.01 (0.96-1.07)	1.08 (1.00-1.15)[b]
Family disposable income				
Higher tertile	80.7 (7.5)	Ref (1.00)	Ref (1.00)	Ref (1.00)
Middle tertile	81.9 (7.6)	0.86 (0.81-0.91)[a]	1.03 (0.98-1.08)	1.17 (1.10-1.23)[a]
Lower tertile	83.1 (8.3)	0.93 (0.87-0.98)[b]	1.04 (0.99-1.09)	1.22 (1.15-1.28)[a]

ORs are adjusted for age at diagnosis and sex. HFRS= Hospital Frailty Risk Score; CCI= Charlson Comorbidity Index; CI=confidence interval; OR=odds ratio; SD=standard deviation. [a]p<0.001. [b]p<0.05.

© 2025 Ding M et al. *JAMA Network Open*.
