# Supplementary material for: Social Factors, Age, and Health at Time of Dementia Diagnosis
Source: JAMA Netw Open. 2025 Feb 21;8(2):e2461117. doi: 10.1001/jamanetworkopen.2024.61117 (PMC11846011; doi:10.1001/jamanetworkopen.2024.61117)
Supplement: Supplement 2. — Data Sharing Statement [file jamanetwopen-e2461117-s002.pdf]

Data Sharing Statement

Ding. Social Factors, Age, and Health at Time of Dementia Diagnosis. *JAMA Netw Open*. Published February 21, 2025. doi:10.1001/jamanetworkopen.2024.61117

Data
Data available: No

Additional Information
Explanation for why data not available: Due to the General Data Protection Regulation in Sweden, the pseudo-anonymized personal data underlying this study cannot be shared publicly. Access to the data and the codes for data analyses can be permitted to external researchers after ethical vetting and establishment of a collaboration agreement. Contact the corresponding author for questions about data sharing (MD).
